# Plain film of the abdomen remains a low sensitivity test in A&E

**DOI:** 10.1007/s11845-023-03427-1

**Published:** 2023-06-21

**Authors:** Daniel P. McKenna, Morgan P. McMonagle

**Affiliations:** Department of Surgery, University Hospital Waterford, Dunmore Road, Waterford, X91 ER8E UK

**Keywords:** Abdomen, Misdiagnosis, PFA, Radiation, X-ray

## Abstract

**Background:**

Plain film abdomens (PFA) are frequently used in the emergency department to help guide the management of patients presenting with abdominal symptoms. A plain film abdomen contributes minimally to clinical scenarios due to low sensitivity and specificity. Is a PFA useful in the emergency setting or does it serve to further complicate decision making?

**Aim:**

We hypothesise that PFAs in the emergency department are over utilised to falsely reassure clinicians and patients alike.

**Methods:**

A search of the National Integrated Medical Imaging System (NIMIS) database in an Irish tertiary referral hospital was conducted. All plain film abdominal radiographs requested by the emergency department between 01/01/2022 and 31/08/2022 were identified. Requests where there was suspicion of foreign body were excluded. A retrospective search of the NIMIS database identified subjects who underwent subsequent imaging.

**Results:**

A total of 619 abdominal films were deemed suitable for inclusion. These comprised of 338 male and 282 female subjects. Subjects had an average age of 64 years. Fifty-seven per cent of PFAs detected no abnormality. Forty-two per cent of subjects had subsequent imaging. The plain film findings correlated with further imaging in only 15% of cases. One case of ruptured aortic aneurysm and 11 perforations were detected on computerised tomography, none of these cases were evident on abdominal X-ray.

**Conclusion:**

Plain film abdomen requests are over utilised in the emergency department. PFAs are not sensitive for detecting acute pathology and should not be used to decide if a patient requires further imaging or a full clinical assessment.

## Background

The plain film abdomen (PFA) is a controversial imaging modality in the surgical patient. PFAs are frequently used in the emergency department to help guide the management of patients presenting with abdominal symptoms. However, the sensitivity and specificity of PFAs make their validity questionable [1, 2]. This poses the question, ‘Is a PFA useful in the emergency setting or does it serve to further complicate decision making?’.

## Aim

We hypothesise that PFAs in the emergency department are over utilised. They may contribute to misdiagnoses through false negative results, thereby missing acute abdominal pathology.

## Methods

A search of the National Integrated Medical Imaging System (NIMIS) database in an Irish tertiary referral hospital was conducted. The Picture Archiving Communication System (PACS) office collated the details of all plain film abdominal radiographs requested by the emergency department between 01/01/2022 and 31/08/2022.

Exclusion criteria consisted of requests with a suspicion of foreign body. Following the implementation of this criteria, PFA requests were analysed examining indication and the final report. A retrospective search of the NIMIS database was used to identify subjects who underwent subsequent imaging. The modality and reported abnormality was noted.

## Results

A total of 638 abdominal films were performed in the emergency department between 01/01/2022 and 31/08/2022. The exclusion criteria were applied, and 619 films were deemed suitable for inclusion. These comprised of 338 male and 282 female subjects. Subjects had an average age of 64 years.

Regarding the PFA reports, 57% detected no abnormality. Suspected obstruction, perforation and abdominal pain were the most common indications given for requesting the PFA. Faecal loading and dilated bowel loops were the most common PFA findings. These were identified in 37% of subjects. In all subjects under the age of 40 who were found to have an abnormality on plain film, none subsequently went on to have higher-order imaging.

Further imaging reports were identified in 42% of subjects. Two hundred twenty-nine had a computerised tomography (CT) scan of abdomen and pelvis filmed, while 49 underwent an ultrasound of their abdomen. The plain film findings correlated with further imaging in 15% of cases. PFAs correctly identified 20 cases of small bowel obstruction, three cases of large bowel obstruction and three cases of sigmoid volvulus. However, they failed to identify 11 intra-abdominal perforations and one case of ruptured abdominal aortic aneurysm that were evident on CT.

## Discussion

Our results highlight the weaknesses associated with PFAs in the acute setting. The low correlation with higher-order imaging and the failure to detect serious intraabdominal pathology are particularly concerning. The Royal College of Radiologists iRefer guidelines propose that PFAs are not indicated for suspected perforation and are of limited benefit in cases of obstruction [3]. It has been shown that closer adherence to these guidelines reduces inappropriate requests and unnecessary radiation [4].

It has previously been reported that a normal PFA does not influence decision making in emergency departments [5]. However, this concept is somewhat confusing. If the normal PFA will be further investigated in the same manner as one with pathology, should the PFA not be abandoned? The low diagnostic yield that PFAs provide deem that they have little influence on which patients undergo further imaging [6]. In this context, it is difficult not to view PFAs as confounders for clinical decision making.

Radiation exposure is another drawback of the PFA. Abdominal plain films expose patients to an average radiation dose of 0.7 mSv, the equivalent of 35 chest X-rays [7]. A CT abdomen and pelvis has an average dose of 14 mSv, with background annual radiation exposure estimated at 3 mSv [7]. The concern with unnecessary radiation exposure is the added risk of malignancy. It is estimated that an exposure to 100 mSv over a patient’s life confers a 1 in 200 risk of radiation-related malignancy [8]. These risks are thought to be more profound in the paediatric population with doses of 20 mSv potentially contributing to neoplasia [9]. These risks need to be considered by every clinician prescribing radiation. It brings into question the indications given for some of the plain films in this study. Is abdominal pain a valid reason for 0.7 mSv of radiation? A PFA should not be the preamble to CT. We propose that it is a needless source of radiation either in patients who require higher-order imaging or in those who need further clinical workup.

Following analysis of the PACS data, the costs associated with the imaging modalities described were investigated. Each PFA cost the hospital in question €80. Regarding the higher-order imaging, an ultrasound abdomen cost €120, a non-contrast CT abdomen and pelvis €180 and a contrast CT abdomen and pelvis €270. In totality, emergency department PFA requests cost the hospital €49,520. This figure would have covered the cost for 275 non contrast CT abdomen and pelvises.

## Conclusion

PFA requests are over utilised in the emergency department. PFAs contribute remarkably little to establishing a diagnosis. PFAs are not sensitive for detecting acute pathology and should not be used to decide if a patient requires further imaging or a full clinical assessment. PFAs are a source of needless radiation exposure and are an unnecessary financial burden.
